# Is Provider Secure Messaging Associated With Patient Messaging Behavior? Evidence From the US Army

**DOI:** 10.2196/jmir.6804

**Published:** 2017-04-06

**Authors:** Vickee Wolcott, Ritu Agarwal, D. Alan Nelson

**Affiliations:** ^1^ Army-Baylor University Graduate Program in Health and Business Administration JBSA Ft Sam Houston, TX United States; ^2^ Center for Health Information and Decision Systems Robert H Smith School of Business University of Maryland College Park, MD United States; ^3^ School of Medicine Stanford University Stanford, CA United States

**Keywords:** patient portal, physician-patient relations, health communication

## Abstract

**Background:**

Secure messaging with health care providers offers the promise of improved patient-provider relationships, potentially facilitating outcome improvements. But, will patients use messaging technology in the manner envisioned by policy-makers if their providers do not actively use it?

**Objective:**

We hypothesized that the level and type of secure messaging usage by providers might be associated with messaging initiation by their patients.

**Methods:**

The study employed a dataset of health care and secure messaging records of more than 81,000 US Army soldiers and nearly 3000 clinicians with access to a patient portal system. We used a negative binomial regression model on over 25 million observations to determine the adjusted association between provider-initiated and provider-response messaging and subsequent messaging by their patients in this population over a 4-year period.

**Results:**

Prior provider-initiated and response messaging levels were associated with new patient messaging when controlling for the patient’s health care utilization and diagnoses, with the strongest association for high provider-response messaging level. Patients whose providers were highly responsive to the messages of other patients initiated 334% more secure messages (*P*<.001) than patients with providers who did not personally respond to other patients’ messages.

**Conclusions:**

Our results indicate that provider messaging usage levels and types thereof predict their patients’ subsequent communication behavior. The findings suggest the need for more study into the factors associated with provider messaging to fully understand the mechanisms of this relationship.

## Introduction

### Background

Better patient-provider communication is important because their relationship is at the center of health care service delivery [1]. A supportive patient-provider relationship has been shown to be associated with many positive outcomes, including increased patient compliance [2], decreased pain [3], and shortened recovery periods [4]. Opportunities to interact with one another are critical to the development of ideal patient-provider relationships [5]. In “Crossing the Quality Chasm,” the Institute of Medicine (2001) [6] recommended the use of phone and email communication between appointments as a visit extender to support a continuous patient-provider relationship [5,7].

Ongoing and expanded communication stands in contrast to the historical, episodic patient-provider relationship that mainly comprises infrequent office visits. Secure messaging could facilitate the development of deeper relationships by increasing interaction time, making patients more comfortable about asking questions and discussing embarrassing issues [8], and allowing physicians to provide better advice and education [9]. However, such benefits are likely to be realized only if patients and providers are both committed users of the technology.

Secure messaging is often provided as part of a patient portal. Unfortunately, studies show that portal use among patients is low, with only 10-32% of patient portal adopters actually using the portal [10,11]. The Centers for Medicare & Medicaid Services recently proposed a change to the Health Information Technology for Economic and Clinical Health Act because providers appear to struggle to engage their patients via electronic means. This recommendation would reduce the current requirement that 5% of patients use secure messaging to simply requiring the presence of the feature [12].

Reducing the communication recommendation may be the wrong approach, given that relatively little evidence has been developed in this area. Researchers have paid surprisingly limited attention to provider and patient usage of secure messaging, and the associated factors are potentially complex. Patients perceive the overall health service quality they receive and develop an associated level of trust and comfort with their clinicians [13], potentially driving behavior such as secure messaging. Patients may lose interest in such resources if providers do not encourage the use of electronic tools or lead by example by becoming active users of the tools themselves [14]. Furthermore, the way and degree to which providers generally engage in messaging may represent a marker of the level of approachability that is perceived by their patients during care. Provider receptiveness to communication, an otherwise difficult-to-assess factor, might be indicated by the willingness of patients to initiate secure messages with their clinicians.

### Aim of This Study

Our associated hypothesis was that providers’ overall messaging behavior might serve as an indicator of their accessibility for or interest in communication, which patients directly or indirectly perceive. Therefore, patients’ use of secure messaging might be related to the extent to which their providers generally use it. Our resulting specific aim was to answer the question: What is the relationship between providers’ past secure messaging types and levels and the initiation of messaging by patients?

We defined provider messaging levels as the extent to which a patient’s primary care provider exchanges messages with his or her other patients, compared with the messaging rates of other providers in the same population. We further distinguished between messages the provider initiated on their own (defined as “provider-initiated messages”) and messages the provider sent in response to patient messages (defined as “provider-response messages”) due to the potentially different causes for these messaging events.

We theorized that provider-initiated messages might be mostly representative of routine operational matters, rather than a personal philosophy or stance on communication that patients could perceive and act upon. For example, such messages could occur due to specific clinical needs including notifications of laboratory or imaging results or reminders. In contrast, choosing to personally respond to patient messages (rather than delegating this function to the supporting clinical team, or even ignoring messages) might represent a stronger commitment to communication. This receptive mindset could be perceived by other patients, creating increased comfort among them for message initiation. Because we had no data on the specific content of messages, controlling for the distinct message types provided an initial method for isolating any difference in impact between them.

## Methods

### Data

We employed messaging data from the Army Medicine Secure Messaging Service (AMSMS) used by the US Army Medical Department (AMEDD), introduced in January 2011. Patients in this health system use AMSMS to securely message their primary care and medical teams to request medical advice, appointments, lab results, referrals, and prescription renewals; record medical information; and access educational materials. Providers initiate messages in AMSMS to send care reminders, appointment reminders, and direct patient messages. AMSMS was rolled out in a consistent manner across Army hospitals and clinics, with a team visiting each location to conduct training and provide system access.

We theorized that AMEDD would be an excellent setting for this study because it is a large, integrated organization providing health care to the Army’s 3.95 million service members, retirees, and family members. We were able to utilize a rich, extensive dataset with over 25 million observations constituting eligible months for health care within the Army’s medical system. The system comprises 8 medical centers, 27 community hospitals, and 180 primary care clinics [15], with common policies and procedures and similar patient populations across medical facilities.

The primary dataset therefore consisted of de-identified administrative, medical, and training data from official military information systems documenting AMEDD care. This repository was established at the University of Maryland Center for Health Information and Decision Systems (CHIDS) as the Military Medical Informatics Data Set (MMIDS). MMIDS contains data on over 820,000 active duty soldiers in total, capturing military service and associated events during January 2011 through December 2014. The data are arranged into a longitudinal record of observed person-months of military service during this time, across which values for the selected variables were free to vary with time. Data elements in the dataset include, among other variables, age, deployment history, time-in-service, rank, race, marital status, body mass index, self-reported health measures, medical diagnoses, medical appointment data, prescription medications, physical fitness test scores, and tobacco use.

We obtained AMSMS usage logs from its implementation in January 2011 through November 2014, and linked the patients in the MMIDS who were portal users with their specific messaging actions. The AMSMS data included 727,951 secure messages for 439,368 patient users, of which 81,645 were active duty soldiers, involving 2983 provider and staff users. We studied active duty soldiers only because MMIDS solely consists of information on these soldiers.

This study was reviewed and determined to be exempt by the University of Maryland Institutional Review Board and underwent secondary review at the Defense Health Agency’s Human Research Protection Office. All statistical analysis was conducted using Stata 13 software (StataCorp).

### Variables

Our dependent variable represented the number of messages, if any, sent by each patient registered to use the AMSMS in each observed person-month. Values for this parameter varied with time within each such month across the longitudinal dataset, where applicable. Our requirement was that qualifying messages were initiated by the patient and did not represent a reply to providers. To control for each primary care provider’s overall AMSMS message rate, we calculated the number of messages which a patient’s provider initiated and responded to other patients in each month (excluding the focal patient). We then divided this value by the number of patients enrolled in AMSMS for that provider. The quotients were then categorized by tertiles into low, medium, and high messaging when compared with the messaging values of all providers in the sample. We note that these measures were exogenous to the patient, and therefore expected to be uncorrelated with individual outcomes. Our approach appeared adequate to control for provider workload because the providers in our sample worked full-time and had approximately equal patient empanelments in accordance with Army policy.

We expected that patients might have been more likely to send secure messages following health care visits and in response to ongoing medical issues. The patient-specific number of health care visits could have been associated with problem severity and chronicity, which in turn could have been associated with an individual increased need for messaging. Therefore, we included health care utilization measures and medical conditions within the previous 3 months as independent variables to eliminate variance explained by these factors.

The International Classification of Disease System, 9th Revision, Clinical Modification (ICD-9) remained in use by the Military Health System at the time of the studied events. We included medical conditions in the following categories defined by ICD-9 codes because we observed these to be the five most prevalent condition types among the active duty Army soldiers in our dataset: musculoskeletal issues, mental health diagnoses, hypertension, sleep apnea, and dyslipidemia. Additionally, we controlled for calendar month and location. Table 1 describes each of the variables.

**Table 1:** Description of variables.

Variables		Variable name	Description
**Independent variables**		
	Recently diagnosed patient medical conditions	
		mentaldx	Whether or not the soldier had a diagnosis of anxiety disorder, adjustment disorder, personality disorder, depression, or post-traumatic stress disorder within the previous 3 months
		mskdx	Whether or not the soldier had a diagnosis of musculoskeletal issue (eg, back injury, joint pain) within the previous 3 months
		sleepapndx	Whether or not the soldier had a diagnosis of sleep apnea within the previous 3 months
		hypertensiondx	Whether or not the soldier had a diagnosis of hypertension within the previous 3 months
		dyslipidemiadx	Whether or not the soldier had a diagnosis of dyslipidemia within the previous 3 months
	Health care utilization measures	
		primecaretot	Number of monthly primary care visits
		ervisit	Number of monthly emergency room visits
		speccaretot	Number of monthly specialty care visits
	Secure messaging factors	
		provinitiatecat	The number of messages the focal patient’s provider initiated to other patients in a month, representing the providers’ messaging level, categorized by tertiles. Message types included are care reminders, appointment reminders, and patient communication. Three categories: low, medium, and high
		provresponsecat	The number of messages in which the focal patient’s provider responded to other patients in a month, representing the providers’ messaging level and categorized by tertiles. Message types included responses to appointment requests, billing questions, lab or test results, doctor notes, referral requests, and prescription refills. Three categories: low, medium, and high
**Dependent variable**		
		patientmsg	The number of messages initiated by each patient in each observed person-month (excludes replies to provider messages)
**Other factors**		
		installation	Patient’s site of military service, one of 32 possible locations
		month	Monthly dummies for time controls

### Analysis

Because the outcome measure was a count variable and was overdispersed, we utilized a negative binomial regression model [16]. We included patient-level fixed effects to control for patient-level heterogeneity that could impact portal usage. We note that the fixed effect is able to account for differences in patient characteristics such as demographics as well as technology acceptance factors idiosyncratic to a patient, such as perceived usefulness of the technology, perceived incompatibility with needs, and so on [17]. Patient-level fixed effects allow for a separate intercept for each patient, controlling for unobserved differences among individuals [16]. The provider messaging categories and health care utilization measures were lagged to the previous month to ensure they occurred before the patient sending the message. As data were reported at the monthly level, it was not possible to distinguish order of events within a month. The regression model was as follows:

log(*patientmsg*_it_) = β_0i_ + β_1_*provinitiatecat*_it-1_ + β_2_*provresponsecat*_it-1_ + β_3_*primcaretot*_it-1_ + β_4_*ervisit*_it-1_ + β_5_*speccaretot*_it-1_ + β_6_[Patient Medical Conditions]_it-1_ + β_7_*installation* + β_8_*month* + ε_it_

## Results

### Descriptive Statistics

Tables 2 and 3 provide descriptive statistics of the 81,645 patients who adopted the portal between January 2011 and November 2014. Each month, 7% of patients initiated a secure message. Health care providers initiated on average 0.007 (SD 0.06) messages per patient per month, and responded on average to 0.09 (SD 0.19) messages per patient per month.

**Table 2:** Patient messaging and health care utilization characteristics.

Description	Mean (SD^a^)
Number of patient-initiated messages per month per patient	0.07 (0.38)
Patient primary care visits	0.48 (0.87)
Patient specialty care visits	0.23 (1.39)
Patient emergency room visits	0.01 (0.14)

^a^SD: standard deviation.

**Table 3:** Characteristics of patients who adopted the portal (N=81,645).

Characteristics		Portal adopter^a^, n (%)	
**Gender**			
	Male		64,206 (78.64)
	Female		17,439 (21.36)
**Age category (in years)**			
	18-22		13,496 (16.53)
	23-27		17,652 (21.62)
	28-35		23,187 (28.40)
	36+		27,310 (33.45)
**Education level**			
	High school equivalency		4139 (5.07)
	High school diploma		39,018 (47.79)
	Some college		13,733 (16.82)
	Bachelor’s degree		14,957 (18.32)
	Graduate		9798 (12.00)
**Marital status**			
	Never married		21,367 (26.17)
	Married		54,253 (66.45)
	Divorced		6025 (7.38)
Had a dyslipidemia diagnosis within the previous 3 months (in 3 months before adoption)			2792 (3.42)
Had a hypertension diagnosis within the previous 3 months			727 (0.89)
Had a mental health diagnosis within the previous 3 months			1755 (2.15)
Had a musculoskeletal diagnosis within the previous 3 months			6572 (8.05)
Had a sleep apnea diagnosis within the previous 3 months			1886 (2.31)

^a^Adopters were all patients who signed up for the portal. Not all of them were actual users.

### Main Results

**Figure 1:**
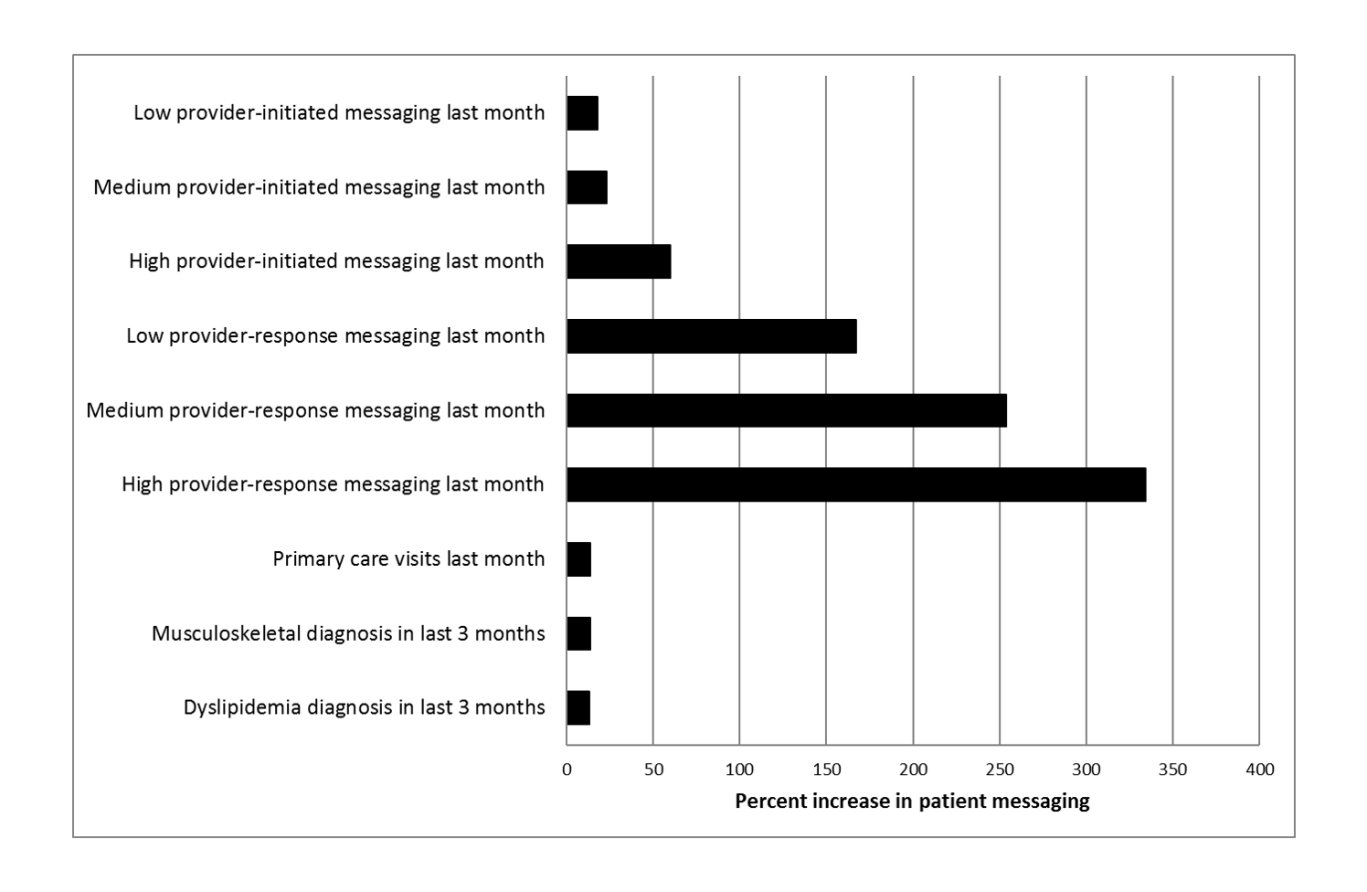
Graph of relative associations of patients’ provider messaging and health care factors with patient messaging.

As displayed in Table 4 and Figure 1, we found that patients receiving care from high response- and high initiation-messaging providers were substantially more likely to initiate a secure message than patients with nonmessaging providers, that is, those providers who did not send a message in the previous month. Patients with high initiation-messaging providers were 60% more likely to send a secure message than patients with noninitiation-messaging providers. Strikingly, patients with high response-messaging providers sent 334% more messages than those with nonresponse-messaging providers. Patient message initiation among low response-messaging providers was 254% higher than among nonresponse-messaging providers. Among medium response-messaging providers, patients demonstrated increased messaging by 167% when compared with nonresponse-messaging providers.

As might be expected, health care utilization and medical conditions also impacted patient messaging. For every additional primary care visit during the month prior, patients sent 14% more messages in a given observed month. Specialty care and emergency room visits in the month prior were not associated with the number of messages a patient sent. Having a musculoskeletal or dyslipidemia diagnosis in the previous 3 months were each associated with statistically significant increases in the number of patient messages by 14% and 13%, respectively. But, mental health, hypertension, and sleep apnea were not associated with patient messaging habits. For comparison, we conducted regression analysis without including the five medical conditions. Results were largely unchanged, with very modest differences. The incidence rate ratios (IRRs) for high provider-initiated messaging and medium and high provider-response messaging increased by 0.01, and the specialty care visits last month became statistically significant when excluding medical conditions, but only provided an IRR of 1.01. It appeared that when only controlling for specialty care visits, the model failed to adequately address the more specific information provided by selected medical conditions.

**Table 4:** Regression results. The model included time controls, location controls, and patient fixed effects.

Variables		Patient-initiated messages		
		IRR^a^	95% CI	*P* value
**Monthly provider-initiated messaging**				
	No provider-initiated messaging last month	1.00	Referent	
	Low provider-initiated messaging last month	1.18	1.12-1.24	**<** *.001* ^b^
	Medium provider-initiated messaging last month	1.23	1.17-1.30	**<** *.001*
	High provider-initiated messaging last month	1.60	1.51-1.70	**<** *.001*
**Monthly provider-response messaging**				
	No provider-response messaging last month	1.00	Referent	
	Low provider-response messaging last month	2.67	2.53-2.82	**<** *.001*
	Medium provider-response messaging last month	3.54	3.37-3.73	**<** *.001*
	High provider-response messaging last month	4.34	4.13-4.55	**<** *.001*
Primary care visits last month		1.14	1.12-1.15	**<** *.001*
Emergency room visits last month		1.02	0.97-1.07	.49
Specialty care visits last month		1.00	0.99-1.01	.21
Musculoskeletal diagnosis within the previous 3 months		1.14	1.10-1.19	**<** *.001*
Mental health diagnosis within the previous 3 months		1.01	0.95-1.07	.76
Hypertension diagnosis within the previous 3 months		1.00	0.92-1.07	.92
Sleep apnea diagnosis within the previous 3 months		1.01	0.96-1.07	.64
Dyslipidemia diagnosis within the previous 3 months		1.13	1.08-1.19	**<** *.001*

^a^IRR: incidence rate ratio.

^b^*P* value in italics indicate statistical significance (*P*<.05).

## Discussion

### Principal Findings

In this study, we demonstrated that among US Army soldiers, increased provider-initiated and provider-response messaging were associated with statistically-significant increases in the adjusted probability of patient-initiated secure messaging. We also demonstrated that provider-response messaging had a much larger impact on patient messaging than provider-initiated messaging. Given our ability to control for health care utilization and medical conditions, the study offers strong evidence that provider messaging usage is a critical, overlooked factor associated with their patients’ behavior.

We can suggest no direct mechanism by which provider messaging with a given patient would impact the decision of another patient to initiate a message. We therefore theorize that patients’ willingness to initiate messages may stem from their appreciation of provider traits that, in turn, are associated with the provider’s propensity to robustly engage in different types of secure messaging. These traits might be more evident among clinicians who choose to personally respond to patient messages and do so at high rates. Provider-response messaging may be more influential than provider-initiated messaging because provider-response messaging is likely a more personal type of messaging, tailored to each specific patient’s needs.

Patients may appreciate the general communicative nature of providers who take the time to respond to messages personally, rather than having a staff member respond. Alternatively, it is possible that patients may initiate messages due to frustration with their ability to communicate with providers because their providers use secure messaging to avoid face-to-face and telephone encounters. Provider messaging use therefore requires substantial further study in order to better understand how providers differ when stratified by secure messaging usage levels and types.

Our findings carry implications for policy addressing the wider diffusion and uptake of critical patient-centered health information technologies. Health information technologies have been heralded as one possible solution to addressing the high cost and often low quality of health services delivery. However, as noted, authorities may reduce the pressure on providers to engage in patient portal and secure messaging use. Perhaps a more useful approach would be to require that providers demonstrate a minimum level of engagement with secure messaging and to sponsor studies that examine the factors associated with all use levels and types.

Additionally, our findings revealed that medical problem types were related to patient message initiation in the studied population. Health care organizations may therefore expect increased secure messaging from patients following primary care visits and recent diagnoses of certain conditions, such as musculoskeletal issues and dyslipidemia. Organizations that are at early stages of implementing secure messaging resources might need to ensure that health care teams and patients in care settings in which these problems predominate are notified of the potential for high messaging rates. These settings might also provide the greatest opportunities for the study of secure messaging and the emergence of associated best practices.

### Limitation

A limitation of this study is that the findings may not be broadly generalizable because we studied a younger, more male population than that seen in the general public, and this population is preselected for health as a requirement of military service.

### Future Work

Further study in other groups will be required to assess the external validity of our findings. However, we note that the patient portal software, including secure messaging capability used by the military, is the same software used in many civilian health care settings. The ability to verify or refute our findings in large civilian medical systems should therefore be feasible in future research employing data from this or similar systems.

### Conclusions

This was the first study to use a large, robust dataset to empirically investigate provider messaging behavior and its potential relationship with the willingness of patients to send secure messages. New data will be needed to address the potential, unobservable factors that explain our main finding of a provider-patient usage association. Candidate data might include new surveys assessing patient perceptions, supporting a study comparing those cared for by clinicians with varying secure messaging use levels and types. In preparation for such research, we will leverage the datasets employed for this study that include large reserves of additional information on patient trajectories and provider behavior. We expect these data to provide new insights with our ongoing research to better understand the impact of patient and provider utilization of technologies and the factors associated with these critical contributors to health.

The project also revealed relatively high outpatient health care utilization (Table 2) for a generally young population of individuals who were preselected for health in order to serve. As the military is an environment with universal, free health care, this finding suggests the potential to study care utilization behavior in such an environment. We will assess this study concept for feasibility as part of our ongoing review of the substantial data resources available to the research team.

## Abbreviations

AMEDD: US Army Medical Department
AMSMS: Army Medicine Secure Messaging Service
CHIDS: Center for Health Information and Decision Systems
IRR: incidence rate ratio
MMIDS: Military Medical Informatics Data Set
